# Cytomegalovirus Colitis Masquerading as a Recurrent Colonic Polyp in a Patient With Neurofibromatosis Type 1

**DOI:** 10.14309/crj.0000000000001587

**Published:** 2025-01-21

**Authors:** Colin P. Slaymaker, Bhargavram Channagiri Srinivas, Pranay Reddy, D. Matthew Shoemaker, Andrea E. Stand, Ryan K. Rader

**Affiliations:** 1 Department of Medical Oncology, University of Kansas School of Medicine-Wichita, Wichita, KS; 2 Department of Medical Oncology, Gadag Institute of Medical Sciences, Gadag, Karnataka, India; 3 Department of Gastroenterology, University of Kansas Medical Center, Kansas City, KS; 4 Department of Infectious Disease, University of Kansas Medical Center, Kansas City, KS; 5 Department of General Internal Medicine, University of Kansas Medical Center, Kansas City, KS; 6 Department of Medical Oncology, University of Kansas Medical Center, Westwood, KS

**Keywords:** cytomegalovirus, colitis, colonic polyp

## Abstract

Cytomegalovirus colitis most commonly affects immunocompromised patients, although it is a rare cause of gastrointestinal bleeding in immunocompetent patients. Older age, chronic disease, and critical illness are also important risk factors and may lead providers to consider the diagnosis in otherwise immunocompetent patients. Endoscopic presentation is variable and does not significantly influence outcomes. Although most immunocompetent, noncritically ill patients improve with or without antiviral treatment, mortality rates are as high as 71.4% in critically ill patients. Such mortality rates necessitate that intensive care providers remain wary of the diagnosis in any patient presenting with gastrointestinal bleeding. We present a rare case of cytomegalovirus colitis masquerading as a recurrent colonic mass in a patient with neurofibromatosis type 1.

## INTRODUCTION

Associated with a benign course in most immunocompetent patients, cytomegalovirus (CMV) may cause end-organ disease leading to significant morbidity and mortality on reactivation in immunocompromised patients.^1^ CMV colitis is the most common form of CMV reactivation disease, often presenting nonspecifically with symptoms including diarrhea, hematochezia, fatigue, fever, and weight loss.^1,2^ Most commonly associated with immunocompromising conditions such as HIV, CMV colitis may also afflict “immunocompetent” patients, although many of these patients are likely at least somewhat immunocompromised due to older age, the presence of multiple chronic comorbidities, or the presence of acute illness.^3^ In fact, CMV reactivation is relatively common in critically ill patients, with studies showing 20%–40% rates of active infection in patients admitted to the intensive care unit.^4^ Furthermore, previous studies of immunocompetent patients diagnosed with CMV colitis found that 79.1% of the population had at least 1 major comorbidity with a median age of 68 years among the group.^3^

Diagnosis of CMV colitis is best made through endoscopy with biopsy, which most commonly demonstrates discrete ulcers or diffuse erythema localized to the colon.^3^ Though ulcerative and exudative appearances are most commonly seen on endoscopy, the condition may present with variable morphology and at any location between the esophagus and the anus.^3^ We present a rare case of CMV colitis manifesting as a colonic mass at the site of a previous bowel resection in a patient with neurofibromatosis type 1 (NF1).

## CASE REPORT

An 80-year-old woman with NF1 presented to the emergency department in June 2024 after an episode of hematochezia. Before the development of these symptoms, she had a subacute clinical decline in her health with weight loss, memory loss, and decreased ability to independently perform activities of daily living. This functional decline was most likely multifactorial, with chronic abdominal pain and depression contributing to decreased oral intake and the patient's inability to perform activities of daily living. In 2015, she was diagnosed with a gastrointestinal stromal tumor (GIST), treated with partial gastrectomy and small bowel resection with Billroth II gastrojejunostomy. No recurrences of her GIST had been reported before admission. In 2022, the patient was found to have an inflammatory fibroid polyp of the cecum after routine colonoscopy with biopsy and subsequent resection of the terminal ileum and proximal colon with ileocolic anastomosis. Family history was significant only for cancer in the patient's son, although she was unaware of the specific type.

On presentation, her vital signs were within normal limits. Generalized abdominal tenderness was present on examination, although the abdomen was soft and nondistended. The patient received a loading dose of pantoprazole 80 mg intravenously and was continued on pantoprazole 40 mg by mouth twice daily for empiric treatment of potential upper gastrointestinal bleed. Her home anticoagulants and antihypertensives were discontinued. A complete blood count demonstrated a hemoglobin level of 10.3 g/dL and no leukocytosis. An abdominal and pelvic computed tomography scan demonstrated a lobular mass at the ileocolic anastomosis. She was subsequently admitted for gastroenterology consultation and further workup. She reported persistent discomfort in her midabdomen, radiating to the back, and intermittent nausea, but was otherwise asymptomatic with no further episodes of hematochezia.

**Figure 1.**
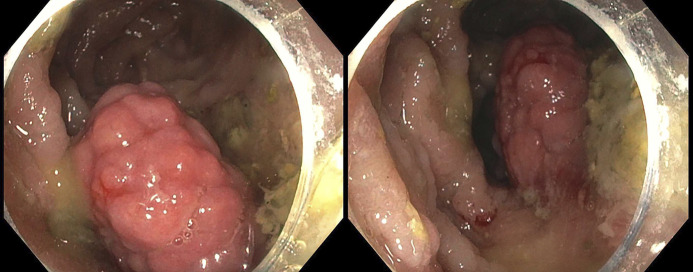
Colonoscopy images showing a nonobstructing, frond-like, fungating, and polypoid mass at the ileocolic anastomosis, measuring 5 cm in the largest dimension.

The patient's hemoglobin level dropped to 8.8 g/dL on the second day of admission but soon returned to 10.0 g/dL and remained stable thereafter. She underwent esophagogastroduodenoscopy with colonoscopy on the third day of admission. Esophagogastroduodenoscopy demonstrated 2 small friable polyps at the gastrojejunal anastomosis without additional significant findings. Biopsy of these lesions demonstrated ulcerated tissue with reactive changes and granulation tissue but no evidence of malignancy. Colonoscopy revealed a nonobstructing, frond-like, fungating, and polypoid mass at the ileocolic anastomosis, measuring 5 cm in the largest dimension (Figure 1). The mass was biopsied, with high suspicion for malignancy at that time given her histories of NF1, GIST, and inflammatory fibroid polyp of the colon. Surgical oncology was consulted and informed the patient that no surgical options were available because of her comorbidities.

**Figure 2.**
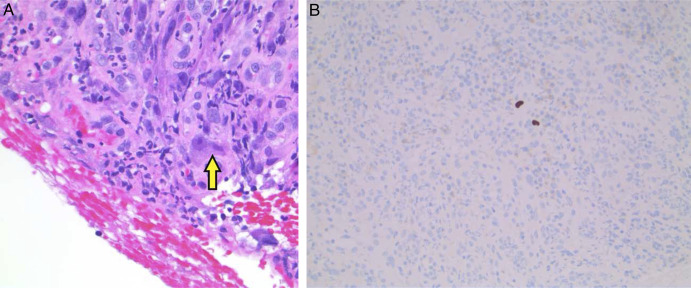
Histopathology specimens demonstrating the classic “owl's eye” appearance of CMV nuclear inclusions (A, yellow arrow, 200× original magnification, hematoxylin-eosin) and positive CMV immunostaining (B). CMV, cytomegalovirus.

The biopsy revealed ulcerated granulation tissue without evidence of malignancy. Interestingly, the presence of “owl's eye” inclusions was noted on histologic examination; CMV immunostaining was positive (Figure 2). Subsequent serum studies demonstrated CMV viremia with a level of 32 IU/mL on CMV DNA polymerase chain reaction, positive IgG antibodies to CMV, and negative IgM antibodies to CMV. HIV combined antigen/antibody testing was nonreactive. Infectious disease was consulted and started ganciclovir 5 mg/kg IV every 12 hours. The patient tolerated ganciclovir therapy well, remaining afebrile and reporting improvement in her abdominal pain after treatment initiation.

Displaying clinical improvement and no adverse reactions to antiviral therapy, she was discharged and transitioned to valganciclovir 900 mg by mouth twice a day for a duration to be determined on an outpatient basis based on her response. The patient was scheduled for outpatient follow-up with infectious disease and recommended to undergo a repeat colonoscopy after completion of therapy to assess for resolution of the mass. However, she continued to have multifactorial functional decline and elected to forgo additional evaluation, transitioning to hospice care in July 2024. The patient unfortunately died soon after her transition to hospice care.

## DISCUSSION

CMV colitis is an important cause of morbidity and mortality in immunosuppressed patients and those with critical illness. Commonly presenting with diarrhea and hematochezia, the diagnosis is often delayed, given that these symptoms can mimic those of other infectious colitides or an inflammatory bowel disease flare.^1^ In immunocompetent patients, CMV colitis most commonly presents with discrete ulcers or diffuse erythematous lesions, either of which may be associated with exudate, on endoscopy.^3^ However, the presentation of CMV colitis as a distinct, exophytic mass is uncommon.

Appropriate evaluation of any elderly patient presenting with hematochezia and a colonic mass on imaging should include colonoscopy. However, distinguishing gastrointestinal malignancy from CMV colitis based on gross appearance is challenging. Previous studies demonstrate a mortality rate as high as 71.4% in critically ill, immunocompetent patients with confirmed CMV colitis, even when receiving appropriate antiviral therapy.^5^ Outcomes are much better in noncritically ill, immunocompetent patients, with mortality rates of 9.3% and 38.4% of patients improving without antiviral therapy.^3^

In the intensive care unit setting, CMV viral load, IgG, and IgM could serve as useful adjuncts for diagnosis and even as indications to begin treatment in such high-risk patients while awaiting biopsy results. However, these studies do not localize the site of end-organ disease and are less sensitive than biopsy with histopathologic evaluation, which is needed to confirm the diagnosis.^3^ Furthermore, given the much lower rates of mortality in noncritically ill patients, serological tests should not be used as indications to begin antiviral therapy.

Although CMV colitis is most common in immunocompromised patients, the case patient had no easily identifiable risk factor predisposing her to such disease manifestations. In such patients, HIV assays should be performed with antiretroviral therapy initiated if positive. However, it is important to remember that many “immunocompetent” patients presenting with CMV colitis may have some extent of impaired immunity because of older age or the presence of chronic comorbidities such as diabetes mellitus, chronic kidney disease, and coronary artery disease.^3^ For instance, previous research has shown that both mucosal and secretory immune function in the gastrointestinal tract declines with age.^3^ As such, it is crucial that providers be wary of more “inconspicuous” forms of immunocompromise and consider the diagnosis in all patients presenting with gastrointestinal bleeding, particularly the elderly.

Especially in critically ill patients, whose rates of mortality from CMV colitis are much higher, it is imperative that providers perform CMV serological testing, perform CMV stains on histopathological specimens, and consider empiric antiviral treatment on the development of gastrointestinal bleeding regardless of the endoscopic appearance of any identified lesions. In the case patient, it is likely that her older age, history of coronary artery disease, and overall frailty contributed to the development of CMV colitis. There is no evidence that patients with NF1 are more prone to viral infection.^6^

Although there is no consensus on the need for antiviral therapy in immunocompetent patients with CMV colitis, all critically ill patients with biopsy-confirmed CMV colitis should promptly be started on intravenous ganciclovir with weight-based dosing, given the potential for significant morbidity and mortality.^3^ In more stable patients, providers should make the decision to treat on a case-by-case basis taking into consideration the patient's comorbidities, age, and functional state. Endoscopic appearance and localization do not affect outcomes and should not influence the decision to begin antiviral therapy.^3^ The dose should then be titrated based on patient tolerability and continued in an intravenous formulation until the patient is discharged, at which time the patient should be transitioned to oral valganciclovir.

Given the rarity of CMV colitis presenting with a mass lesion, colonoscopy should be performed soon after discharge to ensure resolution of the mass and definitively rule out the possibility of underlying neoplasia. Although the case patient did not undergo this workup given her transition to hospice, this evaluation is essential to confirming the diagnosis. If a mass is present on repeat colonoscopy, it should be biopsied at multiple sites with repeat CMV immunostaining performed. Antiviral therapy should continue for an extended period on an outpatient basis with discontinuation based on clinical and endoscopic resolution.

It should be noted that the patient's transition to hospice, subsequent nonadherence to a full course of therapy, and lack of follow-up colonoscopy limit the authors' ability to provide recommendations regarding long-term follow-up of immunocompetent patients who develop CMV colitis presenting with a mass lesion. In addition, further studies evaluating the efficacy of antiviral treatment in immunocompetent adults with CMV colitis are needed.

## DISCLOSURES

Author contributions: CP Slaymaker penned the manuscript with the help of BC Srinivas. DMS provided the included images. P. Reddy, DM Shoemaker, AE Stand, and RK Rader played pivotal roles in the patient's care, suggested avenues to explore in the discussion, and revised previous drafts of this report. CP Slaymaker is the article guarantor.

Financial disclosure: None to report.

Previous presentation: This case was presented as a poster abstract by Dr. Pranay Reddy at the American College of Gastroenterology Annual Scientific Meeting on October 28, 2024, in Philadelphia, PA.

Informed consent was obtained for this case report.

## References

[1] Azer SA Limaiem F. Cytomegalovirus colitis. 2023. In: StatPearls [Internet]. Treasure Island, FL: StatPearls Publishing; 2024 Jan. 31194388

[2] Gupta M Shorman M. Cytomegalovirus. 2023. In: StatPearls [Internet]. Treasure Island, FL: StatPearls Publishing; 2024 Jan.

[3] Yoon J Lee J Kim DS Endoscopic features and clinical outcomes of cytomegalovirus gastroenterocolitis in immunocompetent patients. Sci Rep. 2021;11(1):6284. 33737711 10.1038/s41598-021-85845-8 PMC7973552

[4] Papazian L Hraiech S Lehingue S Cytomegalovirus reactivation in ICU patients. Intensive Care Med. 2016;42(1):28–37. 26424680 10.1007/s00134-015-4066-9 PMC7095171

[5] Siciliano RF Castelli JB Randi BA Vieira RD Strabelli TM. Cytomegalovirus colitis in immunocompetent critically ill patients. Int J Infect Dis. 2014;20:71–3. 24406737 10.1016/j.ijid.2013.11.008

[6] Wei CJ Gu SC Ren JY The impact of host immune cells on the development of neurofibromatosis type 1: The abnormal immune system provides an immune microenvironment for tumorigenesis. Neurooncol Adv. 2019;1(1):vdz037. 32642666 10.1093/noajnl/vdz037 PMC7212924

